# Nano-Clays for Cancer Therapy: State-of-the Art and Future Perspectives

**DOI:** 10.3390/jpm12101736

**Published:** 2022-10-19

**Authors:** Francesca Persano, Stefano Leporatti

**Affiliations:** 1 Department of Mathematics and Physics, University of Salento, 73100 Lecce, Italy; 2 CNR Nanotec-Istituto di Nanotecnologia, 73100 Lecce, Italy

**Keywords:** nano-clays, cancer therapy, delivery systems, nanocarriers, halloysite, montmorillonite, kaolinite

## Abstract

To date, cancer continues to be one of the deadliest diseases. Current therapies are often ineffective, leading to the urgency to develop new therapeutic strategies to improve treatments. Conventional chemotherapeutics are characterized by a reduced therapeutic efficacy, as well as them being responsible for important undesirable side effects linked to their non-specific toxicity. In this context, natural nanomaterials such as clayey mineral nanostructures of various shapes (flat, tubular, spherical and fibrous) with adjustable physico-chemical and morphological characteristics are emerging as systems with extraordinary potential for the delivery of different therapeutic agents to tumor sites. Thanks to their submicron size, high specific surface area, high adsorption capacity, chemical inertia and multilayer organization of 0.7 to 1 nm-thick sheets, they have aroused considerable interest among the scientific community as nano systems that are highly biocompatible in cancer therapy. In oncology, the nano-clays usually studied are halloysite, bentonite, laponite, kaolinite, montmorillonite and sepiolite. These are multilayered minerals that can act as nanocarriers (with a drug load generally between 1 and 10% by weight) for improved stabilization, efficient transport and the sustained and controlled release of a wide variety of anticancer agents. In particular, halloysite, montmorillonite and kaolinite are used to improve the dissolution of therapeutic agents and to delay and/or direct their release. In this review, we will examine and expose to the scientific community the extraordinary potential of nano-clays as unique crystalline systems in the treatment of cancer.

## 1. Introduction

Although cancer is currently the most studied human disease, it still represents an unresolved challenge, affecting a large portion of the world’s population. Approximately 18.1 million new cases of cancer are diagnosed annually, and 9.6 million people die from this disease—mainly, metastatic cancers [1,2]. While the treatment of non-metastatic cancers has a considerable chance of success, surgery and radiotherapy are treatments that cannot be cured once the cancer has spread throughout the body [3]. Cytotoxic chemotherapeutics and hormonal antagonists are currently the therapeutic strategies adopted in the treatment of metastatic neoplasms [4,5]. However, these drugs are characterized by a non-specific mechanism of action, with the indiscriminate destruction of both tumor cells and healthy cells [6,7]. Furthermore, a prolonged administration of these drugs often leads to the development of the so-called tumor multidrug resistance (MDR), with a significant increase in the costs of therapy and a waste of medical resources [8,9]. From this arises the need to develop innovative therapeutic strategies which are targeted for improved efficacy and safety [10,11]. Sub-micron-sized, high-surface-area nanoparticles (NPs) allow for the specific targeting and elimination of cancer cells, with improved selectivity in cancer treatment [12]. In the last thirty years, important advances have been made in the realization of innovative nanoformulations (such as the development of stable colloidal nanoformulations of low-water-solubility drugs) that have radically changed approaches in the treatment of cancer [13]. The research to date has focused on the design and development of nanocarriers with desirable chemical-physical characteristics in order to obtain longer circulation times, delayed elimination, an increased accumulation in the desired site and the possibility of a controlled release of the load through an external or internal stimulus [14,15]. Furthermore, thanks to additional functions, nanomaterials can be applied to allow for the co-administration of different therapeutic agents—for the selective targeting to tumor cells and the penetration through biological barriers (such as the Blood Brain Barrier) [16,17]. Consequently, nanomaterials constitute a valid alternative for improving the efficacy of conventional anticancer drugs thanks to an improved bioavailability, specificity and safety profile [18,19]. The extraordinary potential of nanoparticles in cancer therapy is determined by their unique properties, such as their nanoscale size, high surface area and highly adjustable surface chemistry [20]. Nanoparticles can be produced from both organic and inorganic materials or a combination of them. Among organic nanoparticles, we find dendrimers and lipid-based and polymer-based nanoparticles [21]. While among the inorganic nanoparticles, we find gold, silver, silica, calcium carbonate, calcium phosphate, quantum dots and carbon-based nanoparticles. Among the inorganic materials, we also find clay minerals, which derive from the chemical erosion of sedimentary rocks and are characterized by a stratification on a nanometric scale [22,23]. Clay minerals are particularly abundant on earth; as a result, humans have been collecting this material for thousands of years, using it in medical formulations. Many centuries ago, the Egyptians were among the first to develop medicines from clay minerals [24]. Subsequently, Galen, a famous physician of Ancient Greece, produced terra Sigillata, which is a set of shaped and imprinted red earth tablets (most likely montmorillonite or kaolinite enriched with iron oxide) [25]. Nowadays, clay minerals have aroused the interest of the scientific world as vectors in the treatment of cancer due to their biocompatibility, grain size, morphology, high specific surface area and high charge density [26,27].

**Figure 1:**
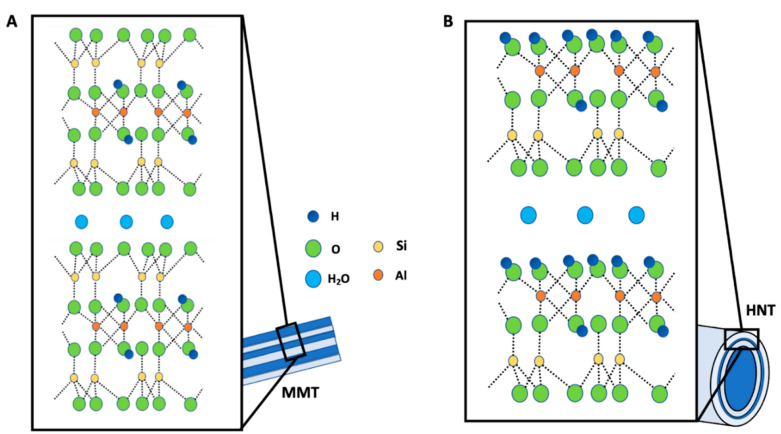
Chemical structures of MMT (**A**) and the HNT (**B**).

With this work, we will summarize the literature describing the potential of nano-clays in the treatment of cancer, with particular attention to the formulations based on halloysite nanotubes (HNT) and montmorillonite (MMT) [28]. On the basis of the mineralogical composition, about 30 different types of nano-clays with different properties are distinguished [29]. Nano-clays are fine-grained crystalline materials whose structural unit is represented by a layer, and these layers tend to arrange themselves on top of each other [30]. The individual layers are represented by tetrahedral and/or octahedral sheets, and it is precisely the arrangement of these sheets that plays a key role in the classification of clay minerals. At the level of the tetrahedral sheet, the silicon-oxygen tetrahedra are connected to the neighboring tetrahedra, sharing three angles; the fourth corner of each tetrahedron will instead become part of the adjacent octahedron sheet [31]. The octahedral sheet is mainly made up of aluminum or magnesium in six-times coordination with the oxygen of the tetrahedral sheet and with the OH [32]. Hydrogen bonds, van der Waals forces or electrostatic forces are the main factors that unite the different layers with the formation of parallel sheet stacks [33]. This stacking results in regular empty spaces between adjacent layers, defined as inter-layers or galleries, and are accessible to organic polar liquids, organic cations and water [34]. Clay minerals can be classified as 1:1, 2:1 or 2:1:1. A 1:1 clay structural unit consists of a tetrahedral sheet and an octahedral sheet [35]. A 2:1 clay has a structural unit consisting of an octahedral sheet sandwiched between two tetrahedral sheets. Finally, a 2:1:1 clay has a 2:1 base layer structure characterized by an octahedral sheet adjacent to an octahedral sheet fused between two tetrahedral sheets [36]. Halloysite is a nanotube formed by several layers of alumosilicate, consisting of tetrahedral sheets of SiO_4_ and octahedral sheets of AlO_2_(OH)_4_ (Figure 1A). Montmorillonite is a phyllosilicate with a 2:1 layer structure (1 nm-thick), whose structural unit is characterized by two tetrahedral silica sheets and one octahedral alumina sheet (Figure 1B) [37].

**Figure 2:**
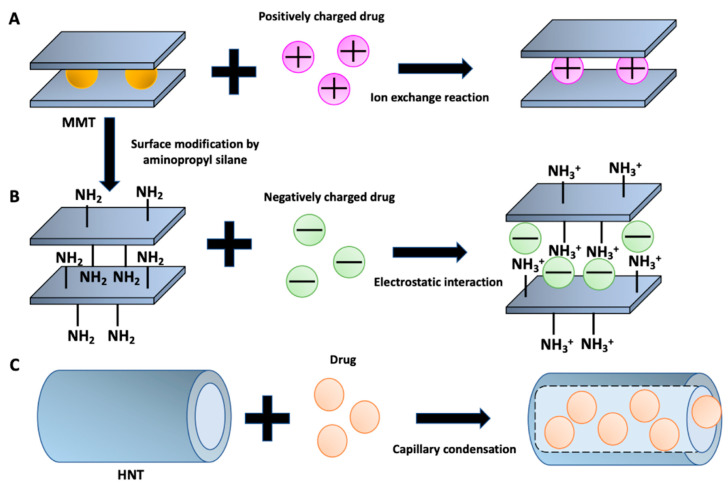
Schematic representation of the interaction of MMT (**A**)/modified MMT with aminopropyl silane (**B**) and HNT (**C**) with a drug.

The binding/interaction of drug molecules with clay materials involves several chemical and physical interactions, including cation exchange, hydrophobic affinity and electrostatic interaction [38]. The charge and the charge density of clayey nanomaterials play a key role in the adsorption and binding processes of the different therapeutic agents; the charge on the drug molecules can bind through electrostatic interactions [39]. Thus, the negatively charged layers of montmorillonite, through the ion exchange reaction, can interact with positively charged therapeutic agents (Figure 2A). Furthermore, the montmorillonite surface can be suitably modified with aminopropyl silane for the loading of drugs with a negative charge (for example, telmisartan and flurbiprofen) (Figure 2B) [40]. Furthermore, the loading mechanism of the therapeutic agents in the tubular structure nanoclay is different from that of the plate structure nanoclay. Halloysite has a tubular structure in which drugs can be incorporated into the tubular pore by capillary condensation (Figure 2C) [41]. These nanotubes can act as nanocontainers for the efficient entrapment and release of different classes of therapeutic agents. In order to ensure a prolonged/controlled release of drugs, halloysite nanotubes can be coated with different polymers such as dextrins [42].

## 2. Key Properties of Nano-Clays for Application in the Delivery of Therapeutic Agents

Clay minerals are often used in the pharmaceutical industry, either as net or activated ingredients. The complex of chemical-physical characteristics of clay nanomaterials makes these nanostructures suitable for use in the controlled loading and release of different classes of therapeutic agents, including anticancer drugs [43]. Clay minerals are able to interact with the molecules of different therapeutic agents, but they can also interact with the different additive constituents (such as polymers) that can be used in the production of nano-formulated medicines with optimal properties [44]. There are many published studies regarding the use of nano-clays (including MMT, HNT and kaolinite) as nanocarriers for the administration of anticancer drugs [45]. These nano-formulations based on nano-clays can be used for the development of tablets for oral administration or can be suitably modified for the development of systems suitable for the targeted administration and controlled release of various anticancer agents [46]. In this regard, an interesting strategy is the development of nanocomposites with clayey mineral NPs in polymeric matrices for the realization of nano systems with improved pharmaceutical characteristics compared to the single constituents [47]. The intensity of the therapeutic effect of a drug depends primarily on the concentration of the drug itself at the target site. Therefore, in drug therapy, it is essential to ensure optimal levels of the therapeutic agent at the target site (or at least in the blood) and to maintain them for the duration of treatment [48]. In addition, it is desirable to minimize what may be the temporal variations in the concentration of the therapeutic agent with the use of an appropriately modified administration nano system to avoid periods of underdose or overdose [49]. It follows that pharmaceutical products evolve through modifications of the administration nanocarriers—modifications that determine a variation in the speed and time of release at the site of action compared to conventional drugs [50]. The main factors that have a significant impact on the effective success of nano-clays in application as administration systems will be summarized below.

### 2.1. Size of the Nano-Clays

**Figure 3:**
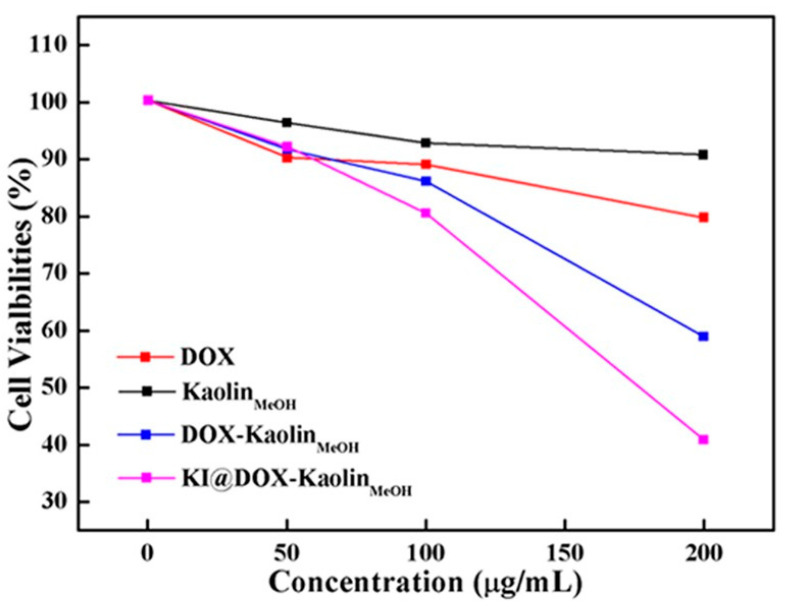
Evaluation of the cytotoxic activity of the different nanoformulations by MTTassay. Papillary thyroid carcinoma cell line TPC1 was incubated for 24 h with free DOX, KaolinMeOH, DOX-KaolinMeOH and KI@DOX-KaolinMeOH at different concentrations. Adapted from ref. [53].

Nano-clays can have different morphologies, including flat, film or tubular, and a thickness ranging between 1 and 200 mm. However, it is known that, for nano-clays, the optimal size for their use as nanocarriers in drug delivery is up to 200 nm [51]. Different-sized nano-clays find different applications—for example, doxorubicin-intercalated kaolinite (DOX), with a diameter between 400 and 500 nm, revealed pH-sensitive behavior and antitumor activity towards ten tumor cell cultures [52], while intercalated kaolinite with DOX has a diameter between 150 and 200 nm and has demonstrated an antitumor activity against papillary thyroid cancer (Figure 3) [53].

In addition, the particle size affects the drug release. In one study, magnesium aluminum silicate (MAS) was employed for the entrapment of propranolol (PPN), a β-blocking agent, and release kinetics were related to particle size and other factors [54]. The results obtained by the authors demonstrated that the pH of the preparation of the complexes has no impact on the release of PPN, while one of the most important factors influencing the release of the drug is precisely the size of the particles.

### 2.2. Nano-Clays’ Pore Size and Volume

Other important factors for the application of nano-clays in nanomedicine are the size of the pores and the volume of the pores. The trapping or loading efficiency of the different therapeutic agents is mainly influenced by the size of the pores and cavities (especially for HNT) of the nano-clays [13,16]. To increase the DOX drug loading efficiency, the spacing of the kaolinite nanoparticles (NPs) was increased from 0.72 to 4.16 nm with the intercalation of different organic species at different chain lengths [52]. In another study, the distance between the adjacent layers of kaolinite was widened from 0.72 to 0.85 nm through the intercalation of methoxy molecules, without changing the morphology. This made it possible to achieve a DOX carrying capacity of 54.52% by weight [53].

Drugs or therapeutic agents (such as enzymes) can be loaded into the HNT lumen. In HNT, the carrying capacity is strongly influenced by the lumen diameter; as the lumen diameter increases from 10–15 to 30–40 nm, in turn, the carrying capacity increases, which even reaches 30–40%, i.e., similar to that of synthetic polymer microcapsules [41].

### 2.3. Charge and Zeta Potential of Nano-Clays

The different types of clay nanomaterials are characterized by different charge densities—for example, the MMT and the surface of the HNTs are predominantly negatively charged. The negative charge of the surface of the nano-clays is mostly at physiological pH; moreover, at these pH values, the clayey nanomaterials used had a large specific surface area of about 57 m^2^g^−1^, suggesting an important potential of the nano-clays for the extended binding of positively charged drugs [55]. Furthermore, the negative charge of kaolinite and its intercalation compounds have provided sites for binding through electrostatic interaction with the positively charged DOX [52]. HNTs have a positively charged lumen, whereby anionic molecules (such as DNA) can be trapped in the lumen of the nanotubes, while the outer surface of the HNTs is negatively charged at most pH settings, allowing for several changes [56].

### 2.4. Loading Efficiency of the Nano-Clays

The loading efficiency is one of the most important parameters of NPs with respect to the use of them as nanocarriers in the administration of anticancer agents [57]. A low loading efficiency in polymeric NPs of hydrophilic drugs was a limitation for the realization of a drug delivery nano system, as well as a high porosity, resulting in a rapid release of the load [58]. Water-insoluble polymers as well as clay NPs have allowed these limits to be overcome thanks to an improved loading efficiency and greater load release efficiency. Loading efficiency is the measure of the percentage of therapeutic agents loaded into the NPs compared to the total weight of the NPs [59]. In clay NPs, a high load of the therapeutic agents was obtained thanks to the entrapment of the molecules in the lumen of the nanotubes using retarding polymers through cationic coating or by exchanging the water of the interlayers with low-molecular-weight agents in order to obtain a moderate speed of release of the load [60]. Composite irinotecan microspheres based on MMT and sodium alginate (AL) were developed in one study. Following the entrapment of irinotecan (Ir) in MMT, the hybrid obtained was composed with AL, obtaining Ir-MMT-AL nanocomposite microspheres through the technical method of ionotropic gelation. The composite microspheres were then tested in vitro in a simulated intestinal fluid (pH 7.4, at 37 °C) with the aim of determining whether the composite beads produced could constitute a valid alternative to conventional chemotherapy in the treatment of colorectal cancer. The results obtained clearly demonstrated that MMT and MMT together with AL are effectively able to control the release of Ir, making it sustained, with a reduction in the amount of the drug released and in the rate of release [61].

An interesting strategy turned out to be the use of polymer composites of clayey materials (CPC) in order to modify the release of the load. The development of CPC allows for the improvement of the properties of the single constituents—those of clay particles (such as the stability of dispersions of clay particles) and those of polymers (including mechanical properties, swelling capacity and cell uptake). In this regard, in a recent study, MMT particles were intercalated in polylactic glycolic acid in order to produce systems for the administration of Docetaxel (anticancer drug). In vitro, the study of the Docetaxel release profile from these composite NPs demonstrated a sustained release in 25 days. In addition, the presence of MTT improved the cellular uptake of NPs by Caco-2 and HT-29 cells, with a prolonged therapeutic effect of the drug [62].

### 2.5. Biocompatibility

**Figure 4:**
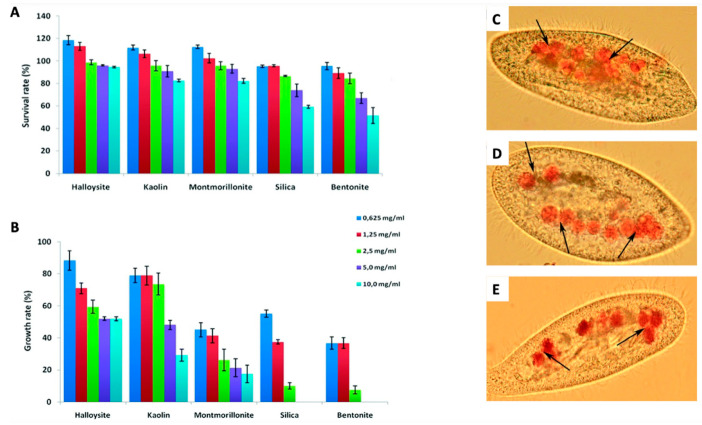
Acute toxicity study (**A**,**B**) and optical microscope images of Congo red-stained food vacuoles in P. caudatum (**C**–**E**). Survival rate (**A**) and growth rate (**B**) of P. caudatum cells following treatment for 24 h with increasing concentrations of NPs. Intact cell (**C**) and cells fed with HNT (**D**) and MMT (**E**). Food vacuoles are indicated by black arrows. Adapted from ref. [67].

One of the fundamental properties for the application of nano-clays in the biomedical field, such as the administration of the various anticancer agents, is biocompatibility. To date, unfortunately, the biological mechanism of the degradation of these aluminosilicates in the human body is not yet understood; it follows that such systems cannot find application as injectable vectors for the delivery of drugs, as they could cause thrombotic events [63]. Several studies have been published on the cellular biocompatibility of HNT, MMT and kaolinite. In most cases, the nano-clays were found to be nanomaterials with almost no toxicity to the different types of mammalian cells. The group of Lvov et al. reported that the electrostatic interactions between HNTs and the cell wall determine an inhibition of the entry of clay NPs into cells, with the prevention of cell damage [64]. For nano-clays, no eco-toxicity has yet been demonstrated; the HNTs have been found to be biocompatible for yeast cells [65], for Escherichia coli (bacterium) [66] and for Paramecium caudatum (freshwater ciliate) (Figure 4) [67].

A high biocompatibility for Caenorhabditis elegans (soil nematode) at high doses (100 mg/mL) has also been reported for HNTs [68]. Regarding the in vitro cytotoxicity of HNTs, it has been studied in various tumor cell lines such as HeLa, MCF-7, A549 and NIH-3T3, with extremely promising results [56]. For MMT, cytocompatibility was tested following short- and long-term cell proliferation, examining the integrity of the cell membrane and studying oxidative stress. In a paper, it was reported that MMT can cause cytotoxic effects at high doses following prolonged exposure; on the contrary, no notable toxicity was reported for mice treated up to the highest concentration tested (1000 mg/kg). It has also been shown that MMT can be absorbed into the body within 2 h, without significant accumulation in any specific organ [69]. These results confirm the safety of MMT for the realization of a sustainable oral delivery system. In vitro, the cytotoxicity of MMT was tested using the Caco-2 cell line, and the results obtained demonstrated the safe nature of MMT. The group of Depan et al. developed hybrid nano systems based on sodium MMT and chitosan-g-lactic acid. After the entrapment of sodium ibuprofen, the effect of treatment with the nanohybrids produced on the cell proliferation of a culture was evaluated for fibroblasts, confirming their biocompatibility [70]. the biocompatibility of the clayey mineral kaolinite was evaluated by Zhang et al. with respect to ten cancer cell lines, including pancreatic cancer, prostate cancer, lung cancer, stomach cancer, esophageal cancer, breast cancer, cancer of the cervix and hepatocellular cancer. The cell viability rate was higher than 85% for most cell lines; that of esophageal cancer cells reached 99.8% and that of the lung cancer cell line was only 61.3% [52]. Previously, Zhang et al. had always tested the cytotoxicity of kaolinite NPs against a papillary thyroid tumor cell line, also demonstrating the biocompatibility of kaolinite in that case [53].

## 3. Nano-Clays in the Treatment of Cancer

Cancer biology is extremely complex and can only be explained through a deep understanding of the role of individual cell types within the tumor, as well as the surrounding Tumor Microenvironment (TME) which, in turn, plays a key role in defining distinctive features [71]. TME is the result of an interaction between different cells such as cancer cells and stromal cells, as well as non-cellular components, such as proteins [72]. It is precisely the extreme complexity of cancer biology that is the main cause of the lack of efficacy for most of the conventional anticancer therapies [73]. Therefore, understanding TME offers an important weapon for the development of intelligent nano systems that are capable of providing a safer and more efficient tool for administering drugs to the tumor site. In this scenario, nano-clays are emerging as systems with extraordinary potential in cancer therapy—not only for their use as vectors for the delivery of different anticancer agents but also for an intrinsic antitumor activity that has been demonstrated for some types of nano-clays [74]. Standard chemotherapeutics used in conventional anticancer therapy have a narrow therapeutic index, are characterized by a low bioavailability and have non-specific activity, in addition to requiring multiple dosages and the use of high dosages. In this regard, the use of nano-clays for the targeted administration of anticancer agents would allow for the reduction of off-target side effects and improve the therapeutic activity of these drugs [45]. The anticancer agents can be trapped in the nano-clays, with the realization of a nano system for the delivery of these agents. All of this allows, compared to classic therapeutic protocols, for a targeted administration of the therapeutic agent to cancer cells, with the improved bioavailability of the drug and a reduction in adverse side effects thanks to the modification of the load release rate [75]. At the same time, the trapping of drugs within the nano system improves the stability of the therapeutic agent, protecting drugs from premature degradation within the body [51]. However, for the efficient use of nano-clays in the delivery of anticancer agents, it is necessary to improve several of their properties, including the specific surface area, type of exchangeable cations, porosity, surface chemistry and zeta potential. In this regard, the nano-clays can be subjected to various modifications, such as the combination with polymers [29]. In the following paragraph, the use of nano-clays—in particular, HNT, MMT and kaolinite in the treatment of cancer—in an uncontaminated form or as systems for the administration of different anticancer agents will be treated.

### 3.1. HNT in the Administration of Anticancer Agents

The reduced toxicity, the extended specific surface area, the curved surface and the ability to interact with the molecules of different therapeutic agents, both through surface adsorption and through an ion exchange reaction, in addition to the solubility in aqueous solutions, make HNT a promising candidate as a system for the delivery of anticancer agents [76]. Halloysites are characterized by different morphologies, including plaques, spheroidal tubes, short tubes and, usually, elongated tubes. HNTs are characterized by a porous tubular structure; the pores can have a size between 2 and 50 nm (mesopores) and even a size greater than 50 nm (macropores) [77]. These unique characteristics make HNTs an extremely versatile system for their application for the delivery of anticancer drugs. HNTs are characterized by two types of hydroxyl groups (OH), external OH groups and internal OH groups, which can be exploited for their functionalization and for drug trapping [78]. The internal lumen of the HNTs can be exploited for the entrapment of the different classes of anticancer agents. It is often necessary to modify the inner lumen and outer surface of HNTs for their use as delivery systems. Furthermore, the efficient release of the therapeutic load and improved drug binding are achieved through the chemical modification of naturally occurring HNTs [79]. Among the different strategies used for the chemical modification of the surface of HNTs before the entrapment of therapeutic agents is the modification with 3-aminopropyltriethoxysilane (APTES). APTES is an organosilane often used for the functionalization of surfaces due to its ease of use and reduced toxicity. APTES allows for the introduction of silanol groups, which will bind (by hydrogen bond) to the OH groups present on the surfaces of the HNTs, acting as an intermediary for the binding of the different drugs. In this regard, ibuprofen was trapped in HNTs and HNTs modified with APTES; the study of the release kinetics from the two nano systems revealed a better release profile and an increased ibuprofen loading efficiency for APTES-modified NPs compared to the unmodified system [80].

**Figure 5:**
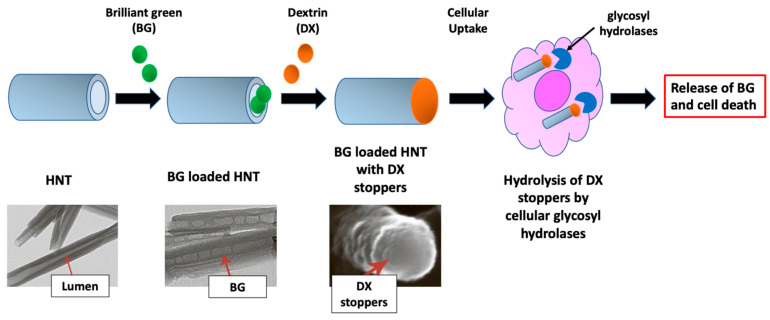
BG-loaded HNT and the application of DX tube end caps for a release of BG activated by intracellular glycolysis hydrolases. Adapted from ref. [83].

HNTs have found an application as vectors for the administration of various anticancer agents. Gemcitabine (GEM) is the primary drug used in the treatment of non-small cell lung cancer (NSCLC). The GEM (analogue of pyrimidine) has a phase-specific action, killing, above all, the cells that are in the phase of DNA synthesis (phase S) and blocking, in some circumstances, the progression of cells from phase G1 to phase S. GEM is usually administered intravenously, but its action is non-specific and has inefficient biodistribution. To overcome these limitations, a solution is represented using HNTs, which can transport GEM across cell membranes via different pathways (via clathrin-dependent or caveolae-dependent pathways). In a study, it was shown that HNTs loaded with GEM can block the cell cycle in A549 cells (adenocarcinoma human alveolar basal epithelial cells), with a reduction in the percentage of S-phase cells. Therefore, HNTs loaded with GEM are able to determine the inhibition of the cell division and growth of A549 cells [81]. In another study, DOX was entangled in HNTs, and the water dispersion of the nano formulated drug compromised the cellular organization of A549 cells. Furthermore, the HNTs allowed for a sustained release of the drug for 2 weeks, without recording an initial burst release [82]. Again, the bright green (BG) anticancer drug was loaded into the HNTs, and the ends of the nanotubes were sealed with physically adsorbed dextrin (DX) caps. The proposed system demonstrated an enzyme-activated BG release, with an accumulation of the preferential drug in highly proliferating A549 cells (Figure 5) [83].

Camptothecin (CPT) (topoisomerase-I inhibitor) is a drug used in the treatment of metastatic colorectal cancer (CRC). CPT is characterized by a reduced solubility in water, as well as presenting toxicity to non-tumor tissues. To overcome these limitations, HNTs have been applied as delivery systems for the release of CPT. Recently, the group of Dramou et al. trapped CPT in the inner lumen of magnetic HNTs (MHNTs) modified with chitosan oligosaccharide (COS) and functionalized with folic acid (FA). The developed nanocomposite therefore exhibited magnetic properties. Furthermore, this composite was subsequently loaded with CPT and the study of the release profile showed a prolonged release of the drug for 60 h. In addition, the release rate of CPT at the acid pH (pH 5) of TME was greater than that at pH 6.8 and pH 7.4. The composite nano system also determined a significant inhibition of the growth of Caco-2 cells (human colon carcinoma cells) and highlighted a specificity in preferentially targeting tumor cells thanks to an improved cellular uptake mediated by FA and COS [84]. Two other drugs with potential in the treatment of CRC are atorvastatin and celecoxib. In the work proposed by Li et al., through a microfluidic technique, the HNTs were encapsulated in a pH-sensitive hydroxypropylmethylcellulose acetate succinate (HPMACS) with the development of composite microspheres (nanotubes-in-microspheres) for the oral co-administration of the two drugs. The microspheres produced exhibited pH-sensitive degradation behavior, with a slightly acidic load protection (pH ≤ 6.5) and rapid release into a simulated intestinal medium (pH 7.4). In addition, the composite microspheres improved the permeability of the drugs, as well as their inhibitory action on the proliferation of colon cancer cells [85].

Gastric cancer still has an important impact on a global level; an innovative therapeutic strategy has been developed to improve the anticancer activity of DOX. The DOX was loaded into the HNTs; then, the DOX-HNTs were encapsulated in soy phospholipids (DOX-HNTs-LIP). The LIP shell has carried out a protective action against DOX-HNT, preventing their direct contact with charged blood ions (such as sodium and chloride) and thus inhibiting the triggering of thrombotic events. Therefore, the nanocomposite has shown a high hemocompatibility thanks to the protective function performed by the LIP. Furthermore, the study of the release behavior of DOX in vitro has shown that the nanocomposites have a pH-sensitive release property, with an accelerated release at acid pH (pH = 5.4). Finally, the MTT assays and the in vivo studies in mice carrying gastric cancer revealed that the nanocomposites had a greater inhibitory activity on the growth of MCF cells than on that of free DOX. In addition, the survival time of the tumor-bearing mice treated with the nanocomposite was increased compared to the control group [86].

There are several challenges for the clinical application of DOX, such as its cardiotoxicity, limited plasma half-life and non-specificity. These limits can be overcome thanks to the use of nano systems targeting tumor tissue. Wu et al. loaded DOX into PEGylated HNT (PEG) and FA functionalized (HNT-PEG-FA) as a nano system for the targeted treatment of breast cancer. Drug release studies have shown a sustained release over 35 h at acidic pH (pH = 5.3) from HNT-PEG-FA. In addition, in vitro cytotoxicity studies have confirmed the ability of HNT-PEG-FA to inhibit cell proliferation and induce apoptosis in MCF-7 cells that overexpress the FA receptor (FR). A reduced cytotoxicity of HNT-PEG-FA was highlighted towards L02 cells (cells lacking FR). Furthermore, in vivo studies performed on 4T1 mice (breast cancer carriers) have highlighted the potential of DOX-loaded HNT-PEG-FA nano systems to inhibit tumor growth, and they have shown that HNT-PEG -FA are able to reduce the cardiotoxicity of DOX compared to that observed for free DOX. Again, HNT-PEG-FA showed a greater accumulation in tumor tissue than other tissues (such as the heart, kidney, spleen and lung) [87].

Osteosarcoma (OSA) is the most common type of bone cancer and usually affects children and young adults. Currently, while considering the significant progress of research in the treatment of OSA, it is necessary to identify new innovative treatments. HNTs have been used as nano systems for the administration of anticancer drugs, such as methotrexate (MTX), aimed at treating OSA. To achieve a controlled release of MTX, the HNTs were alternately coated with polyelectrolytes, positively charged polyvinylpyrrolidone (PVP) and negatively charged poly (acrylic acid) (PAA). In vitro release studies have shown that polyelectrolyte coatings are indeed capable of delivering sustained drug release over 160 min. In addition, the HNT/MTX complexes were able to inhibit the proliferation of OSA cells, resulting in potential delivery systems for the treatment of OSA [88].

Several studies have also shown that HNTs are excellent candidates for the administration of curcumin (CUR), a natural bioactive compound, thanks to the ability of these nano systems to increase its oral bioavailability [89,90]. The group of Liu et al. designed an innovative HNT-based nano system, coated with chitosan (CH), in order to reduce toxicity and provide greater colloidal stability in the bloodstream. First of all, the OH groups on the surface of the HNTs were replaced by carboxylic groups (COOH) with the use of succinic anhydride. Hence, CH was grafted onto HNT-COOH to reduce toxicity, increase the serum stability of HNTs and increase the trapping efficiency of CUR. CH-coated HNTs (HNT-CH) demonstrated an improved loading efficiency (90.8%) and improved drug-carrying capacity (3.4%) compared to pristine HNTs. In addition, the HNT-CH nanocomposite showed a good serum stability without showing obvious haemolytic effects. In vitro cytotoxicity studies revealed a specific toxicity of CUR-loaded HNT-CHs against several tumor lines (including MCF-7, SV-HUC-1, EJ and HeLa), with the highest antitumor activity in the comparisons of EJ cells (human bladder cancer cells) [91]. CUR was also loaded with a high efficiency into HNT-cellulose composite hydrogels prepared by epichlorohydrin crosslinking at elevated temperatures. These composite hydrogels showed a high loading efficiency (21%) compared to the uncontaminated cellulose hydrogels (17%); this is due to the fact that the composite hydrogels are formed by two components, cellulose and HNT, both characterized by a high-capacity adsorption. The CUR release studies showed a controlled release of the drug by the HNT-cellulose composite hydrogels, with the maximum release within 20 h (62.1%). Finally, the cytotoxicity studies have demonstrated the good biocompatibility of the HNT-cellulose composite hydrogels in MC3T3-E1 and MCF-7 cells, as well as the high inhibition capacity towards MCF-7 cells for the HNT-cellulose composite hydrogels loaded with CUR [92].

Another natural compound loaded into the inner lumen of HNTs is polyphenol resveratrol (RES), which, like CUR, has shown promising therapeutic potential as an anticancer agent. However, the use of RES is hindered by its high instability and reduced solubility in water [89]. RES was trapped in the internal lumen of HNTs with the aim of improving its bioactivity and preventing its rapid metabolism. The HNTs were coated using the layer-by-layer (LbL) technique, alternating the protamine salt polyelectrolytes (cationic) and dextran sodium sulphate (anionic). Coating the outer surface of the HNTs with polyelectrolytes resulted in a controlled release of the RES, with a slow and steady release over the 48 h interval. In vitro toxicity studies on MCF-7 cells revealed that HNTs coated with polyelectrolytes and loaded with RES have a high cytotoxicity towards MCF-7 cells, with the induction of apoptosis [93].

Another strategy used in the treatment of cancer is represented by gene therapy, which involves the administration of exogenous nucleic acids inside the cells, with the aim of influencing gene expression. Naked nucleic acids are characterized by a hydrophilic nature, high molecular dimensions and extremely instability, due to their rapid degradation in vivo by nucleases. For years, viral vectors have been the main vectors for the administration of nucleic acids thanks to their ability to transfer genes within human cells [94]. However, high risks are associated with viral vectors, such as important immunogenicity and the possibility of giving rise to insertional mutagenic events, with a consequent alteration in the expression of proto-oncogenes or tumor suppressor genes. All these risks have pushed towards the development of non-viral vectors, which, compared to viral vectors, are characterized by better biosafety, limited immunogenicity and simplified preparation procedures [95]. Considering the considerable size and negative charge of nucleic acids (usually, the surface of inorganic NPs used as vectors in gene therapy), it is functionalized with groups that confer a positive charge to allow for the formation of complexes with nucleic acids. In several studies, clay NPs have shown promising potential as non-viral gene vectors [96]. Molecules widely used in anticancer gene therapy are antisense oligodeoxynucleotides (ODNs) and small interfering RNA (siRNA). These nucleic acids have a remarkable ability to suppress the expression of oncogenic factors. ODNs are short single-stranded DNA molecules consisting of 15 to 30 nucleotides, which, when administered into the cytoplasm, can bind to complementary regions of a target messenger RNA (mRNA), resulting in the inhibition of gene expression. In one paper, Shi et al., in order to facilitate the loading and administration of the ODNs, functionalized the HNTs with APTES. So, the ODNs were labeled with fluorescein for subsequent intracellular tracking. The results obtained showed a good capacity for intracellular administration (98.7%) of the ODNs for the complex, as well as the ability to improve the antitumor potential of the ODNs towards HeLa cells [97]. As for siRNAs, they are small endogenous non-coding RNAs made up of about 21 nucleotides, and they have the ability to inhibit the expression of specific target genes. In this regard, Wu et al. have synthesized modified HNTs for the delivery of anti-survival siRNA in pancreatic cancer cells (PANC-1), with the aim of reducing the levels of the survival protein, which is able to inhibit apoptosis and stimulate the proliferation of cancer cells. For this purpose, the HNTs were modified with the PEI polymer through LbL electrostatic assembly. Subsequently, to highlight the transfection, CdSe quantum dots coated with mercaptoacetic acid were linked by a non-covalent bond (electrostatic interaction) to the anti-survival siRNA to then be linked to the PEI-HNT complexes. The resulting complex showed considerable transfection efficiency in PANC-1 cells (95.6%). In addition, in vitro cytotoxicity studies have shown an increase in apoptosis and an increase in the antitumor potential of anti-survival siRNA. In addition, Western blot analysis, after a 72 h treatment with the complexes, revealed a 90% reduction in target protein levels (survival) in PANC-1 cells. In conclusion, these results confirm the ability of multifunctional complexes based on HNTs to silence the surviving gene, with a consequent reduction in the survival of PANC-1 tumor cells [98]. In a recent work by the group of Long et al., HNTs grafted with a poly (amidoamine) dendrimer (PAMAM) were produced for the intracellular administration of siRNAs, which target the gene-encoding vascular endothelial growth factor (VEGF). In vitro toxicity studies conducted in HUVEC and MCF-7 cells revealed a high biocompatibility of the HNT-PAMAM complexes; moreover, the HNT-PAMAM/siRNA complexes showed a high efficiency of cellular uptake (94.3%), with a 78% reduction in the expression of the mRNA encoded by the target gene. The significant decrease in VEGF expression led to the induction of apoptosis in MCF-7 cells. Studies of antitumor activity in vivo have reported the ability of the HNT-PAMAM/siRNA complex to reduce tumor volume by 55.1% and inhibit angiogenesis [96]. These results suggest that HNT-PAMAM/siRNA complexes could represent a promising strategy in breast cancer gene therapy.

### 3.2. MMT in the Administration of Anticancer Agents

MTT is widely used as a system capable of modulating drug delivery. MMT is characterized by optimal absorption properties, with absorption sites available at the level of its interlayer spaces, particularly on the edges and on the outer surface [99]. The characteristic plaque structure of MMT guarantees a high surface area, high adsorption efficiency, good cation exchange capacity and reduced toxicity. In addition, the layered structure of MMT allows for the stabilization of drug molecules through electrostatic interactions and allows for a controlled release of the therapeutic load thanks to the exchange of bioactive molecules with other ions present in the biological environment [100]. Therefore, ion exchange can be obtained by incubating the solid substrate in a solution of ionic drugs, while, in biological fluids, it will be the counter-ions that displace the drug from the substrate, with a consequent release of the therapeutic agent in the body [101].

**Figure 6:**
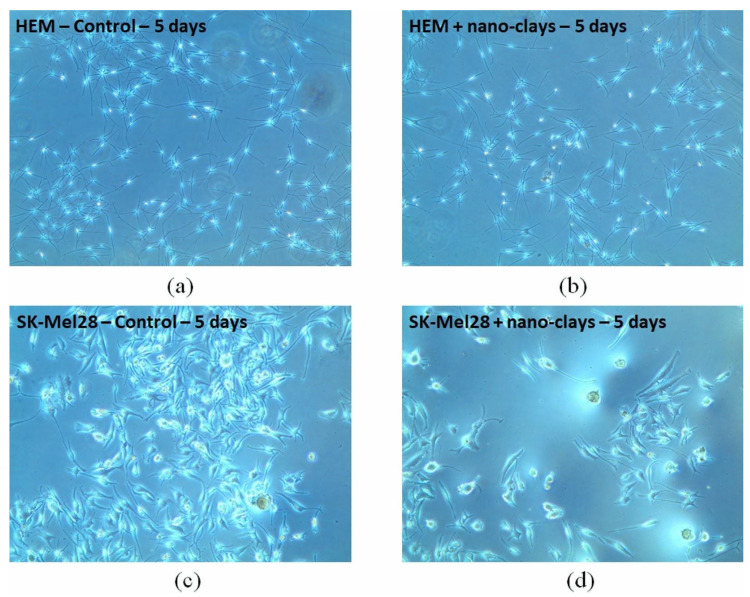
Cell viability of control (untreated) melanocytes (HEM) (**a**) and melanoma (SK-Mel-28) (**c**) after 5 days of incubation; viability of HEM (**b**) and SK-Mel-28 (**d**) cells 5 days after treatment with the nano-clay mixture. Reproduced from ref. [103].

Several studies, in vitro and in vivo, have demonstrated the intrinsic antitumor activity of nano-clays. The uncontaminated clay NPs, thanks to their high specific surface area and surface charge, can modulate the adhesion between tumor cells and the surrounding extracellular matrix, thus preventing metastases [37,102]. In a recent study, Abduljauwad et al. used Na-MMT (Soidum Montmorillonite), Palygorskite and hectorite with the aim of modulating the adhesion of tumor cells to the extracellular matrix. With their results, they have shown that Na-MMT is able to promote cell–cell adhesion, while Palygorskite and hectorite favor cell adhesion to the extracellular matrix in Raji cells (lymphoma cell line). In addition, the combination of Na-MMT with Palygorskite (75:25) resulted in the greatest increase in cell–cell-extracellular matrix adhesions. Another wound healing test conducted with MCF-7 cells revealed that the nano-clays are able to control cell migration, resulting in a delay in gap closure, suggesting that the clay NPs tested could inhibit the migration of cancer cells with the prevention of metastasis formation [102]. In another work, the same combination of Na-MMT and Palygorskite (75:25) of the aforementioned study was used for the control of melanoma metastases. In SK-Mel-28 cells, following treatment with the Na-MMT/Palygorskite mixture, variations in the membrane potential were observed, with an important reduction in cell proliferation and viability in a dose-dependent manner. No significant changes in viability were observed for normal melanocytes, confirming that the Na-MMT/Palygorskite mixture has selective toxicity towards melanoma tumor cells (Figure 6) [103].

Furthermore, the in vivo studies confirmed these results, i.e., the administration of the mixture of nano-clays led to a reduction in tumor size and weight in SK-Mel-28 xenograft mice, as well as an inhibition of mitosis and the induction of necrosis of the cancer cells. This intrinsic antitumor activity of nano-clays is closely associated with the ability of the latter to form adhesions with cancer cells [103]. MMT has also found application in the formation of new hybrid nano systems for the administration of anticancer drugs [104]. In the world, the third most frequent cancer is colorectal cancer (CRC), and the oral route appears to be the preferred route of delivery of anticancer drugs [105]. In this context, pH-responsive nano systems are desirable for the oral administration of anticancer drugs, as they allow for the maximization of the release of the therapeutic agent in the intestinal tract and minimize its early release in the stomach [18]. Paclitaxel is an antimicrotubular agent used in the treatment of several solid cancers, including colon and rectal cancer. However, the limits to be overcome for its clinical application are its reduced solubility in aqueous solvents and low oral bioavailability [106]. In particular, the reduced oral bioavailability is linked to the susceptibility of paclitaxel to the action of cytochrome P450 enzymes present in the intestine and liver. In addition, there are portions of the gastrointestinal tract, such as the intestines, liver and kidneys, with an increased expression of the P-glycoprotein (multidrug efflux pump), also known as the multidrug resistance protein, which contributes to the limited bioavailability of paclitaxel [107]. There have been numerous efforts to develop nanocarriers based on nanoclay for the administration of paclitaxel to cancer cells [108]. In this regard, Dong et al. have created a bioadhesive nano system for the oral administration of paclitaxel. The drug was trapped in poly (D, L-lactide-co-glycolic)-modified MMT NPs (PLGA). The paclitaxel-loaded PLGA/MMT NPs were synthesized through a solvent emulsion/evaporation technique. In vitro paclitaxel release studies from PLGA-MMT NPs revealed a biphasic release profile with an initial burst release followed by a slow and sustained release due to modification with the PLGA copolymer. In addition, cellular absorption studies of PLGA-MMT NPs (loaded with fluorescent coumarin 6) showed an increase in absorption efficiency from 57% to 177% in Caco-2 cells and from 11% to 55% in HT-29 cells. Finally, the nano formulation PLGA/MMT showed an improved residence time in the gastrointestinal tract thanks to the mucoadhesive characteristics of MMT, with the promotion of the oral administration of paclitaxel [109]. In another study, Bothiraja et al. incorporated paclitaxel (cationic) into the interlayer space of Na-MMT via an ion exchange reaction. Subsequently, the drug-loaded MMT was coated with the natural CH polymer. The resulting composite nano system showed controlled paclitaxel release, as well as good biocompatibility. In vitro studies conducted on a human colon cancer cell line (COLO-205) have highlighted the ability of the MMT-CH nanocarrier to improve the anticancer potential of paclitaxel by 1–2 times compared to the non-nanoformulated drug. Functionalization with the CH biopolymer resulted in a reduction of the half-maximum inhibitory concentration (IC50) value of the nano MMT-CH system loaded with paclitaxel [110].

For the chemotherapy of breast cancer, the most deadly and common type of cancer in women, tamoxifen (TMX) (non-steroidal anti-estrogen drug) has been used, which is able to compete with estradiol for binding to the receptor of estrogen. For TMX, the preferred route of administration is oral. Consequently, for effective treatment, it is essential to overcome the limitations that are associated with the oral route of administration, as well as to reduce the side effects of TMX and increase its therapeutic action [111]. Kevadiya et al., for breast cancer therapy, intercalated TMX in Na-MMT interlayers. The resulting TMX-MMT composite was then modified with a hydrophobic polymer, poly-(ε-caprolactone) (PCL), with the formation of a microcomposite system. The microcomposite system showed a controlled release pattern, with a maximum of 72 h, thanks to the presence of the PCL coating. In addition, the genotoxic effect of TMX on a human lymphocyte culture was studied with the comet test; the results obtained showed a reduction in damage at the DNA level when the drug is loaded into the composites by MMT. The effectiveness of the TMX-MMT microcomposite was confirmed by in vitro studies conducted on its HeLa and A549 cells. Furthermore, the in vivo pharmacokinetics (PK) of the hybrid microcomposite were studied in rats after the oral administration of a single dose; the results revealed that the plasma levels of the drug were in the therapeutic window compared to the free drug. The authors concluded that the reduction in genotoxicity is determined by the protection of TMX in the interlayer of the MMT, as well as by the external coating of PCL [112]. Aromatase is a key enzyme in estrogen biosynthesis. An inhibitor of this enzyme is exemestane, which is often used in the treatment of breast cancer. However, exemestane is characterized by a reduced bioavailability linked to its low solubility in water. Furthermore, when exemestane is administered orally, it is susceptible to the first pass effect, with the removal of most of the drug by the liver. With the aim of increasing the oral bioavailability of exemestane, Li et al. have designed an MMT-PLGA nano composite system. The composite nano system showed controlled drug release, with a reduction in initial burst release and a prolonged drug release time. Furthermore, in vitro cytotoxicity studies conducted on MCF-7 cells have shown that composite NPs loaded with exemestane have a greater antitumor potential than the pure drug. In addition, the cytotoxic effect of the nano system loaded with exemestane and the pure drug against MCF-7 cells depended on the concentration of exemestane and the treatment time [113]. In the treatment of triple negative breast cancer, the anticancer drug docetaxel is often used. However, docetaxel is characterized by a reduced solubility in water, and its use is associated with serious side effects which hinder its clinical application. Feng et al. produced a composite nano system, poly (lactide) (PLA)-d-α-tocopheryl polyethylene glycol 1000 succinate (TPGS)/MMT NPs (PLA–TPGS/MMT NPs). The resulting composite nano system was developed for oral administration of the drug docetaxel. The nano composite system loaded with docetaxel was synthesized with a modified solvent extraction/evaporation technique. MMT-PLA-TPGS composite nanocarriers showed enhanced antitumor activity towards MCF-7 cells compared to free docetaxel. In vivo studies with SD rats have shown that the oral administration of the MMT-PLA-TPGS nano system can achieve a half-life of 26.4 times longer than the free drug and improve the oral bioavailability of docetaxel [62].

An anticancer drug frequently used in the treatment of people with pancreatic cancer is GEM. However, this drug is characterized by a reduced plasma half-life (around 15 min) and is rapidly metabolized. Consequently, it is necessary to repeatedly administer high doses of GEM to obtain the desired therapeutic effect; however, this causes significant toxicity and reduced patient compliance. Recently, hydrogel matrices have emerged as an interesting strategy for maximizing therapeutic action while minimizing the adverse side effects of GEM. In this regard, Phan et al. developed a nanobiohybrid hydrogel for the controlled release of the drug GEM. The drug was trapped in the interlayer spaces of the MMT and adsorbed on the surfaces of the MMT NPs. Subsequently, the GEM-MMT NPs produced were dispersed in a biodegradable hydrogel tri-block copolymer poly (ε-caprolactone-co-lactide)-b-poly (ethylene glycol)-b-poly (ε-caprolactone-co-lactide) (PCLA-PEG-PCLA), which is sensitive to temperature. An in vitro drug release kinetics study from the nanobiohybrid hydrogel revealed a significant reduction in initial burst release and sustained release compared to the uncontaminated hydrogel. In vitro toxicity studies conducted on 293T cells with the MTT assay demonstrated that the nanobiohybrid hydrogel exhibits good biocompatibility. In vivo studies on anticancer efficacy conducted on mice carrying pancreatic cancer showed a significant reduction in tumor growth. Therefore, these results suggest that the nanobiohybrid hydrogel may represent a potential system for the controlled release of GEM in pancreatic cancer therapy, where the presence of MMT is essential for efficient entrapment and for a controlled/prolonged release of the drug [114].

The antineoplastic drug 6-mercaptopurine (6-MP) represents an antimetabolite chemotherapy. This is a drug with poor water solubility, and 6-MP rapidly binds to plasma proteins. Due to these characteristics, 6-MP is characterized by a reduced plasma half-life and a low bioavailability. To overcome these limitations, Kevadiya et al. have developed MMT-poly (l-lactide) (PLLA) microcomposite spheres for the oral administration of 6-MP in order to reduce its cytotoxicity and ensure a therapeutic plasma concentration of 6-MP, avoiding periods of overdose or underdose. The anticancer potential of the 6-MP-loaded microcomposite was evaluated in human neuroblastoma cells (IMR32), confirming the ability of the loaded drug to reduce cell viability. The drug release profile study from the MMT-PLLA hybrid microcomposite did not show an initial burst release; instead, it demonstrated a controlled release, with only 22% of the drug released within the first 10 h. In vivo studies conducted in Wistar rats showed that the oral administration of the hybrid microcomposite resulted in an important reduction in drug toxicity, also increasing the mean residence time of 6-MP in plasma. Hence, this MMT-based microcomposite has tremendous potential as a 6-MP dosing system [115].

A natural compound that has shown an important anticancer activity is CUR. However, its rapid metabolic degradation, low water solubility and reduced chemical stability hinder its clinical application. MMT was tested as a potential way to deliver CUR [116]. In this context, hydrophilic biopolymers are gaining particular interest due to their ability to increase the biocompatibility and biodegradability of nano systems. In addition, biopolymers are able to improve the water solubility and colloidal stability in the blood flow of nano systems. Among the various hydrophilic biopolymers, CH is a polymer often used in the functionalization of nano systems due to its mucoadhesive properties and its high biocompatibility and biodegradability. In addition, CH, in acidic conditions, is in an ionized state, making it capable of interacting with cancer cells, as, at acid pH, the CH is positively charged such that it can interact electrostatically with the membranes of negatively charged cells [117]. In a recent study, Khatun et al. have produced MMT-CH nano composite systems for the controlled release of CUR. CUR release studies were conducted at different pH values, demonstrating that, after 6 h, the release rate varied as a function of pH, with an increase in release under acidic conditions (pH = 1.2). The authors suggested that the observed pH-dependent release was a consequence of the different swelling behaviors of CH at different pH values. That is, in an acidic environment, the CH swells easily, and its residual amino groups are protonated, with an increase in the release rate of the CUR. In addition, in vitro toxicity studies confirmed the ability of the composite nano system to reduce the viability of MCF-7 and Hep G2 cells compared to the control (untreated) cells [118].

### 3.3. Kaolinite in the Administration of Anticancer Agents

**Figure 7:**
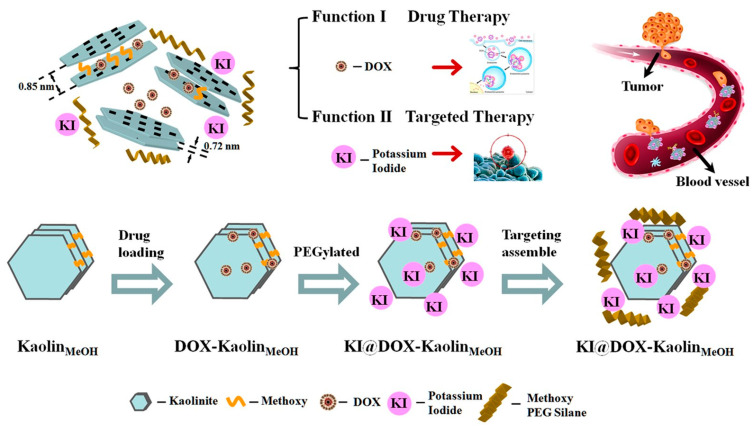
Schematic representation of the synthesis procedure of the methoxy-intercalated kaolinite nanocomposite functionalized with potassium iodide for thyroid cancer therapy. Reproduced from ref. [53].

Kaolinite is the most abundant ingredient in kaolin clay, which is mainly used in the ceramic industry. However, kaolinite has also found several pharmaceutical applications both as an active ingredient and as an excipient due to its unique physicochemical properties [119]. The extraordinary biocompatibility, high biostability and non-immunogenic characteristics make kaolinite a promising candidate as a vector for the administration of drugs [120]. The different products of kaolinite and its derivatives (including ToxiBan^®^) have been indicated for detoxification by the adsorption of ingested toxic substances, such as heavy metals, toxic antibiotic compounds and mycotoxins, with the protection of the mucous membranes and H^+^ contrast [121]. Furthermore, recent studies have shown that nano-clays have intrinsic antitumor bioactivity. In a study, it was shown that the “Kremnevit” kaolinite preparation shows an antitumor potential, resulting in a reduction in tumor mass (by 24%) in mice inoculated with cells of the LLC cell line (Lewis lung cancer) compared to non-cancerous animals. In addition, this increased the rate of superoxide radical formation from intracellular (mitochondria) and extracellular (NADPH oxidase in tumor-associated neutrophils) sources and prevented metastases [122]. This suggests that the “Kremnevit” kaolinite preparation could be a promising material in the reparative procedures of cancer patients. Kaolinite has also found application as a system for the administration of anticancer drugs, such as the drug DOX. Zhang et al. have employed kaolinite for the realization of a bifunctional system to improve the efficacy of administration and, at the same time, reduce the toxicity of the drug DOX in the treatment of thyroid cancer (Figure 7) [53].

**Figure 8:**
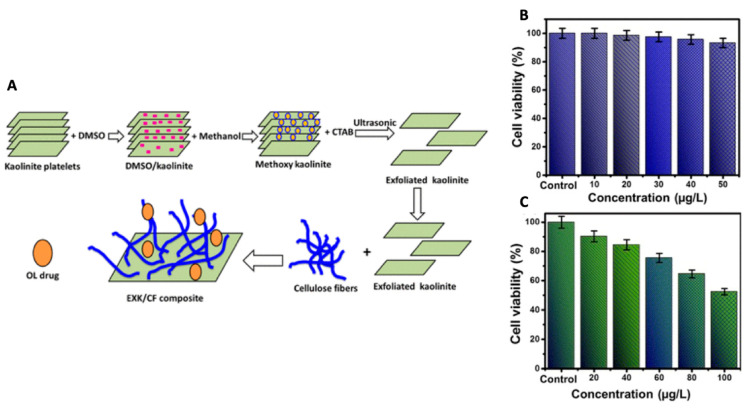
Schematic representation of the synthesis steps of the composite EXK/CF and its subsequent loading with the drug OL (**A**). Cell viability of colorectal fibroblast cells (CCD-18Co) after incubation with the composite EXK/CF (**B**) and of colorectal cancer cells (HCT116) after incubation with the composite EXK/CF loaded with OL (**C**). Reproduced from ref. [123].

First, the expansion of the basal interlayer spacing of kaolinite was achieved by the intercalation of methoxy groups, going from a spacing of 0.72 nm to a spacing of 0.85 nm, thereby increasing the carrying capacity of the molecules and controlling its release speed. The specificity of methoxy-intercalated kaolinite against thyroid cancer was obtained through functionalization with potassium iodide (follicular epithelial cells tend to capture iodine ions for the synthesis of thyroid hormones), and PEGylation has allowed for the improvement of permeability, prolonged the circulation time, improved the anticorrosion of the methoxy-modified kaolinite system in vivo and significantly reduced phagocytosis by macrophages. The study of the drug release from the kaolinite-based nanocomposite has shown a controlled release of DOX sensitive to pH, with an increase in the release rate of DOX in the conditions of a simulated tumor microenvironment (pH = 5.5) compared to normal physiological conditions (pH = 7.4). The system was administered intravenously and as a result of rapidly internalized active targeting. The vector was absorbed by the sodium iodide symporter (internalized via endocytosis) and the drug DOX released at the desired site of action. The MTT viability assay confirmed the reduced cytotoxicity to papillary thyroid cancer cells for methoxy-intercalated kaolinite. For the system loaded with DOX, a dose-dependent therapeutic action was highlighted in vitro. In addition, active targeting played a crucial role in promoting drug accumulation, as demonstrated by the in vivo biodistribution analysis. Thus, the developed kaolinite-based nanocomposite could be used as an effective system for the targeted delivery of DOX in the treatment of thyroid cancer and for targeted drug accumulation at the desired site and reduced toxicity [53]. Different organic compounds, with chains of different lengths (short-chain dimethylsulfoxide (DMSO), medium-chain hexylamine, methanol (MeOH), 3- (APTES) and long-chain dodecyl amine) have been employed in the kaolinite intercalation for one expansion of the interlayer basal spacing. Basal spacing was expanded from 0.72 to 4.16 nm, increasing the carrying capacity and achieving controlled drug release. Cytotoxicity studies have shown that uncontaminated kaolinite and kaolinite intercalation systems are characterized by a high biocompatibility and reduced toxicity towards gastric cancer, prostate cancer, pancreatic cancer, colorectal cancer, cancer of the esophagus, breast cancer and differentiated thyroid cancer. DOX-loaded kaolinite and DOX-loaded kaolinite intercalation compounds showed a faster DOX release at a slightly acidic pH compared to a neutral condition, as well as enhanced (dose-dependent) therapeutic action towards ten different model tumor cell cultures [52]. In a recent study, Tian et al. mixed single layers of exfoliated kaolinite with cellulose fibers, resulting in the production of an exfoliated kaolinite/cellulose fiber (EXK/CF) composite as a vector for the efficient administration of the drug oxaliplatin (OL). A high load-carrying capacity has been demonstrated for the EXK/CF composite system. The study of the release profile of OL from the EXK/CF composite showed a sustained release of up to 100 h, with a maximum release of 86.4 and 95.2%, respectively, in phosphate buffer (pH = 7.4) and acetate (pH = 5.5). Regarding the study of the cytotoxic effect, it revealed a high biocompatibility of the composite EXK/CF towards colorectal cells (CCD-18Co) and a highly toxic effect of the composite loaded with OL towards colorectal cancer cells. (HCT116) (with a cell viability of 31.4%) compared to the free drug (Figure 8) [123]. These results suggest that kaolinite and its intercalation compounds can be effectively used as biocompatible systems for the administration of anticancer drugs for the realization of high-performance nanotherapeutics with superior antitumor potential and reduced side effects.

Table 1 shows the different types of nano-clay used as systems for the administration of the different anticancer agents and their therapeutic effect in the different tumor models.

## 4. Conclusions and Future Directions

The works cited above confirm the extraordinary potential of nano-clays—in particular, HNT, MMT and kaolinite—for the development of systems for the oral administration of various anticancer agents. Such nano systems can release the therapeutic load in a controlled manner, significantly reduce negative side effects, protect therapeutic agents from degradation, increase circulation times, improve the solubility of fat-soluble drugs, reduce renal clearance and promote their interaction with cancerous cells. Furthermore, the ability of clay-based composite systems to incorporate various selective tumor ligands (such as AF and potassium iodide) allows for targeted anticancer therapy, maximizing the concentration of the therapeutic agent at the site of action. The clayey NPs can, among other things, transport proteins and nucleic acids (DNA and RNA), protecting these macromolecules from enzymatic degradation and improving their therapeutic potential. Additionally, the double-charged nature of clay NPs allows for the efficient loading of both positively charged and negatively charged molecules into or onto the surfaces of nano-clays, for which drug molecule charge is not a decisive factor. However, the proper selection of the clay mineral is important for the development of truly therapeutically effective systems. In this regard, the clay minerals present in nature are often suitable for obtaining an effective modulation of drug release, making it essential to use synthetic clay minerals and/or those modified with polymeric additives. In-depth knowledge of the possible drug–clay interactions and of the different release mechanisms is a valuable contribution to the development of clay-based nano systems for drug delivery. The physicochemical properties of clayey NPs, such as the specific surface area, charge (zeta potential), porosity, interlayer space, type of exchangeable cations and hydrophilicity/hydrophobicity can be regulated by modification/functionalization with target polymers and ligands. These modifications are useful for improving the anticancer potential of the loaded therapeutic agent, for a controlled release of the load (ensuring optimal therapeutic levels of the drug at the desired site for the duration of the treatment), for the development of reactive systems to a specific stimulus, for the improvement of the targeted effect and for the reduction of the negative side effects of the drugs. The clinical application of clay-based NPs remains difficult, and further progress is imperative to improving its therapeutic performance. In this regard, it would be useful to consider the physiological changes that occur during carcinogenesis—changes that make the tumor microenvironment unique and different. For example, changes in pH occur, with a tumor microenvironment characterized by a slightly acidic pH. These specific biological alterations can be exploited to develop therapeutic strategies that are sensitive to the specific stimulus, such as the creation of composite systems that use the chitosan biopolymer, which, at acid pH, is positively charged and therefore able to electrostatically interact with the membranes of negatively charged cells. Moreover, at these pH values, the chitosan swells easily with an increase in the release rate of the loaded therapeutic agent [118]. However, the safety of clay-based NPs needs to be further investigated by carrying out pharmacokinetic (absorption, biodistribution, metabolism and excretion) and biopersistence investigations to expand the available preclinical toxicological data and thus promote subsequent clinical studies. The currently available toxicological information generally suggests a risk of thrombotic events following the intravenous administration of the nano-clays, despite their hemocompatibility. Therefore, the data available to date suggest that the oral and topical routes of administration are the most prospective for pharmaceutical formulations based on nano-clays.

## Figures and Tables

**Table 1:** Nano-clays in cancer therapy.

Type of Nano-Clay	Modification	Loaded Antitumor Agent	Tumoral Model	Effect	Reference
HNT	-	GEM	A549 cells (adenocarcinoma human alveolar basal epithelial cells)	cell cycle block, with a reduction in the percentage of cells in the S phase, resulting in the inhibition of cell growth	[81]
	—	DOX	A549 cells	impaired cellular organization	[82]
	ends of the nanotubes sealed with dextrin caps	BG	A549 cells	preferential accumulation of the drug in A549 cells with high proliferating activity	[83]
	modified with chitosan oligosaccharide and functionalized with folic acid (FA)	CPT	Caco-2 cells (human colon cancer cells)	significant inhibition of cell growth and a specificity to preferentially target tumor cells due to improved cellular uptake mediated by FA	[84]
	soy phospholipids (LIP)	DOX	MCF cells (mouse gastric cancer cells) and gastric tumor-bearing mice	the nanocomposites had a greater inhibitory activity on the growth of MCF cells compared to free DOX. The survival time of the tumor-bearing mice treated with the nanocomposite was increased compared to the control group	[86]
	pegylation and functionalization with folic acid (FA)	DOX	MCF-7 cells and mice carrying breast cancer	inhibition of cell proliferation and induction of apoptosis. In vivo tumor growth inhibition and preferential accumulation in tumor tissue, thanks to functionalization with FA	[87]
	coating with polyelectrolytes polyvinylpyrrolidone (PVP) and poly (acrylic acid) (PAA)	MTX	cells of the OSA	inhibition of cell proliferation	[88]
	chitosan	CUR	EJ cells (human bladder cancer cells)	high antitumor activity	[91]
	cellulose	CUR	MCF-7 cells	significant inhibition of cell growth	[92]
	coating with the polyelectrolytes protamine salt (cationic) and sodium dextran sulfate (anionic)	RES	MCF-7 cells	high cytotoxicity towards MCF-7 cells, with induction of apoptosis	[93]
	APTES	ODNs	HeLa cells	improved anti-tumor potential of ODNs towards HeLa cells	[97]
	PEI and CdSe quantum dots coated with mercaptoacetic acid	anti-survival siRNA	PANC-1 cells (pancreatic cancer cells)	increased apoptosis and an increase in the antitumor potential of anti-survival siRNA	[98]
MMT	poly(D, L-lactide-co-glycolic) (PLGA)	Paclitaxel	Caco-2 and HT-29 cells	increased cellular uptake	[109]
	chitosan	Paclitaxel	COLO-205 cells	improved anticancer potential of paclitaxel by 1–2 times compared to the free drug	[110]
	poly-(ε-caprolactone) (PCL)	TMX	HeLa and 549 cells	reduced side effects	[112]
			Wistar rats		
	PLGA	Exemestane	MCF-7 cells	drug-loaded composite NPs have greater anticancer potential than the free drug	[113]
	PCLA-PEG-PCLA	GEM	mice carrying pancreatic cancer	significant reduction in tumor growth	[114]
	poly(l-lactide) (PLLA)	6-MP	IMR32 cells and Wistar rats	reduction in cell viability, while, for in vivo studies, a significant reduction in drug toxicity and an increase in the mean residence time of 6-MP in plasma was observed	[115]
	chitosan	CUR	MCF-7 and Hep G2 cells	reduction in cell viability compared to untreated cells	[118]
Koalinite	Methoxy intercalation, pegylation and functionalization with potassium iodide	DOX	papillary thyroid cancer cells	dose-dependent therapeutic action in vitro and promotion of drug accumulation at the desired site in vivo	[53]
	organic compounds with chains of different lengths	DOX	ten different model tumor cell cultures	enhanced therapeutic action of DOX	[52]
	cellulose fiber	OL	HCT116 cells (colorectal cancer cells)	reduction in cell viability compared to the free drug	[123]

## Data Availability

Not applicable.

## Abbreviations

6-MP	6-mercaptopurine
APTES	3-aminopropyltriethoxysilane
BG	Bright green
CH	Chitosan
COS	Chitosan oligosaccharide
CPC	Polymer composites of clay materials
CPT	Camptothecin
CRC	Colorectal cancer
CUR	Curcumin
DOX	Doxorubicin
FA	Folate
FR	Folate receptor
GEM	Gemcitabine
HNTs	Halloysite nanotubes
LbL	Layer-by-layer
MMT	Montmorillonite
MTX	Methotrexate
NPs	Nanoparticles
ODNs	Antisense oligodeoxynucleotides
OL	Oxaliplatin
OSA	Osteosarcoma
PAMAM	Poly (amidoamine)
PLGA	Poly (lactic-co-glycolic acid)
PLLA	Poly (l-lactide)
RES	Resveratrol
siRNA	Small interfering RNA
TME	Tumor microenvironment
TMX	Tamoxifen
TPGS	Poly (lactide) (PLA)-d-α-tocopheryl polyethylene glycol 1000 succinate
